# Editorial: Neural & Bio-inspired Processing and Robot Control

**DOI:** 10.3389/fnbot.2018.00072

**Published:** 2018-11-08

**Authors:** Ameer Hamza Khan, Shuai Li, Xuefeng Zhou, Yangming Li, Muhammad Umer Khan, Xin Luo, Huanqing Wang

**Affiliations:** ^1^Department of Computing, Hong Kong Polytechnic University, Kowloon, Hong Kong; ^2^Institute of Intelligent Manufacturing, Guangdong Academy of Sciences, Guangzhou, China; ^3^Robotic Autonomy and Collaboration (RAC) Lab, Rochester Institute of Technology, New York, NY, United States; ^4^Department of Mechatronics Engineering, Air University, Islamabad, Pakistan; ^5^Chongqing Institute of Green and Intelligent Technology (CAS), Chongqing, China; ^6^Department of Systems and Computer Engineering, Carleton University Ottawa, Ottawa, ON, Canada

**Keywords:** bio-inspired, robot control, neural processing, processing, intelligent algorithm

## Introduction

The special issue on Neural & Bio-inspired Processing and Robot Control has successfully completed its 3 years of activity. In the beginning, we anticipated that the modern understanding of the biological and neurological system can drive the progress in robotics research in the forward direction. This special issue was started with the aim to promote the inter-disciplinary interaction between bio-inspired systems, and robotics. The profound understanding of both biological and robotics systems will help in bridging the gaps in our understanding of complex problems.

## About the research topic

We are pleased to present 11 research articles, related to robot control, motion planning, and learning. 59 authors from several Asian and European institutions contributed to these articles. The contributions presented in this special issue are focused on the application of bio-inspired insight for the improvement in performance and accuracy of robotic systems. Thus it can be claimed that this special issue plays an important role in the development of a rapidly growing field of research (Cuperlier et al., 2007; Pfeifer et al., 2007; Arbib et al., 2008; Khamassi et al., 2011; Caluwaerts et al., 2012; Li and Zhang, 2018) at the intersection of bio-inspired systems and robotics. In our selection of the research topics, we classified the contributions into two main groups: (a) those applying the insight obtained from the biological systems to improve the performance and accuracy of the control algorithms in robots and (b) those applying the latest developments in neuro-sciences to develop intelligent robotic systems capable of autonomous decision making. We believe that the impact of this research topic can be better described in term of the improvements in robotic systems performance and development of intelligent algorithms by taking inspiration from simple biological mechanisms present in the nature.

One of the novel contribution in this special issue presents an *intention-driven* mechanism to help the severely disabled people in performing daily tasks (Zhang et al.). They used non-invasive brain machine interface to decode the human intentions and developed a robot which can perform the tasks accordingly. Another contribution presents how the learning algorithms can be applied to teach a robot to perform different tasks (Lauretti et al.). Such medical applications of robotics system will greatly help in improving the life quality of patients suffering from severe disabilities. Another similar work uses fNIRS as a non-invasive brain machine interface to control the quadcopter using human thoughts (Khan and Hong.).

Another novel contribution presented in this special issue includes the development of noise tolerant and robust schemes for the robot's motion planning in complicated environments while minimizing the effect of model uncertainties and errors [Ding et al.; Yang et al.; Guo et al.). Besides, another contribution focuses on the theoretical analysis of the genetic algorithms for safety-critical robotic applications and presents mechanisms to prove their safety (Zhang et al.).

Redundancy resolution of redundant robotic manipulators (Jin et al.), motion planning (Xiao et al.; Guo et al.), and control and development of control algorithms to enable the robots to perform human-like postures, gestures, and movements (Tommasino and Campolo) also happens to be a topic of great interest for the contributions in this special issue. Such work will greatly improve the quality of interaction between humans and robots, paving the way for seamless incorporation of robotic system in our daily life.

## Next step

With the tremendous success of the past three Special issues of this Research Topic, we will ask for contributions to these research topics again in next year. We hope that our efforts will contribute to accelerating the robotic research and bridge the inter-disciplinary gaps between the bio-inspired systems, neuroscience, and robotics.

## Author contributions

All authors listed have made a substantial, direct and intellectual contribution to the work, and approved it for publication.

### Conflict of interest statement

The authors declare that the research was conducted in the absence of any commercial or financial relationships that could be construed as a potential conflict of interest.
